# Predicting the Potential Global Distribution of the Invasive Species *Aethina tumida* Murray, 1867, and Its Natural Enemy *Steinernema carpocapsae* (Weiser, 1955)

**DOI:** 10.3390/insects17060541

**Published:** 2026-05-22

**Authors:** Li-Fang Cheng, Yu-Liang Xiao, Cheng Zhang, Jia-Ke Zhang, Yu-Xin Li, Tong-Yin Xie, Qing Zhao

**Affiliations:** 1 College of Plant Protection, Shanxi Agricultural University, Taigu 030800, China; lfc3302@163.com (L.-F.C.);; 2 College of Plant Protection, Northeast Agricultural University, Harbin 150030, China

**Keywords:** MaxEnt and CLIMEX model, invasive pest, ecological niche models, suitable habitat, biological control

## Abstract

Invasive alien species threaten the security of agricultural and natural ecosystems. *Aethina tumida* Murray, 1867, threatens bee colony health and apicultural sustainability. However, the entomopathogenic nematode, *Steinernema carpocapsae* (Weiser, 1955), may be a potential biocontrol agent. Models (MaxEnt and CLIMEX) were used to predict the potential global distribution of both species under climate change. The results indicated that under the current climatic conditions, both models found suitable habitats for *A. tumida* primarily in South America, southern Africa, and South Asia, whereas *S. carpocapsae* exhibited a broader global spread. Notably, CLIMEX predicted a more extensive distribution than MaxEnt for both species. The MaxEnt results indicated that North America, Europe, and central Australia are suitable habitats for *A. tumida* expansion in SSP245 (2050s) and SSP585 (2070s), whereas *S. carpocapsae* was predicted to expand into Asia, North America, and Africa in SSP126 (2090s), SSP245 (2030s), and SSP585 (2070s). The CLIMEX results indicated that under the A1B and A2 climate scenarios, highly suitable habitats for both species decreased significantly, whereas they are predicted to moderately and marginally increase markedly in the 2100s. The potential distribution of *A. tumida* will depend on suitable climatic conditions and the presence of host bees. These results provide a scientific basis and support in preventing or controlling *A. tumida*.

## Simple Summary

This study used two ecological niche models, MaxEnt and CLIMEX, to predict the potential distribution of *Aethina tumida* and *Steinernema carpocapsae*. As an important invasive apicultural pest, *A. tumida* poses a serious threat to global beekeeping production, whereas *S. carpocapsae*, its natural enemy, is a potential biological control. Overall, CLIMEX predicted a wider range of suitable habitats than MaxEnt for both species. Under future climate scenarios, suitable habitats for *A. tumida* may expand further, with particularly prominent risks in Europe and North America where hosts are widely distributed. These findings provide a scientific basis for developing pest monitoring, cross-border quarantine, and control strategies; effectively providing early warning; and preventing global invasion and spread.

## 1. Introduction

Global climate change, particularly rising temperatures and shifting precipitation patterns, has profoundly reshaped the geographical distribution and suitable habitats of species, shifting ranges toward higher latitudes and elevations [1,2]. Temperature is a critical environmental determinant that regulates insect distribution and directly constrains survival, developmental rate, and reproductive potential. Within optimal thermal thresholds, warming can enhance insect growth and population proliferation. However, once tolerance limits are exceeded, heat stress induces physiological disorders, reduces reproductive fitness, and can precipitate population collapse [3]. Simultaneously, invasive insects substantially alter the population dynamics and spatial distribution of indigenous species through competitive exclusion, predation, or pathogen transmission, thereby disrupting established interspecific interaction networks and triggering the irreversible degradation of ecosystem structure and function. Of particular concern is the marked synergy between climate change and anthropogenic activities, including international trade, land use modification, and transportation networks. Elevated temperatures expand the potential habitat range of invasive species, while globalization increases the probability of incidental insect introduction and establishment via commodity movement and transport infrastructure. Consequently, their spread is accelerating, and biodiversity loss and ecosystem disequilibrium are exacerbated [4,5]. Invasive alien species frequently modify the population size and distribution patterns of native species, disrupting regional species interrelationships and subsequently inducing irreversible alterations in the structure and function of invaded ecosystems. Such incursions not only cause substantial economic damage to agriculture, forestry, animal husbandry, and fisheries but also compromise environmental safety and public health [6]. Accordingly, a comprehensive analysis of the complex interactions between invasive insects and local biotic communities, as well as abiotic environmental factors, is imperative to elucidate the ecological drivers underlying outbreak dynamics and develop precise monitoring and early warning systems alongside environmentally sustainable management technologies. Thus, the ecological and economic risks posed by invasive insects may be systematically mitigated [7].

The small hive beetle *Aethina tumida* Murray, 1867, is a destructive, invasive pest of honey bees originating from sub-Saharan Africa, where it is widespread in tropical and subtropical regions [8,9]. It is listed as one of the six most important bee pathogens by the World Organization for Animal Health (WOAH) (https://www.woah.org/en/what-we-do/animal-health-and-welfare/animal-diseases/, accessed on 25 November 2025) [10,11,12]. Propelled by the rapid expansion of the beekeeping industry and increased trade in bee products, this pest has spread to North America, South America, Asia, and Oceania, with reports documented in nearly 20 countries [13,14,15]. Both adult and larval stages feed on the bee brood, honey, and pollen, excavating comb structures and compromising hive integrity. Beetle larvae excrement induces honey fermentation, generating foul odors that precipitate colony absconding [16,17]. Infestations result in discolored, fermented honey that emits a distinctive odor resembling that of rotten oranges. When nest structures and hives sustain damage and fermentation, honey may effervesce through bubbles and leak from the hive. The larvae frequently deposit viscous, malodorous residues, which induce bee abandonment. Adult beetles exhibit a predilection for bee eggs and larvae, which severely compromises colony development and potentially causes colony collapse, absconding behavior, or mortality [18,19]. Given the photophobic behavior of adults, they seek refuge in the corners and crevices upon hive inspection. The detection of adults at the bottom of the hive necessitates vigilance for potential larval damage. Early detection is challenging because the larvae remain concealed within sealed cells [20]. *Aethina tumida* consumes all hive products and disperses through multiple mechanisms, including autonomous flight, the transportation of infected colonies, and human-mediated assistance. Primary dispersal pathways encompass migratory beekeeping operations, including migrating bee colonies, beehives and beeswax; soil attached to various kinds of import–export packaging; and parasitism of circulating fruits and vegetables [21,22,23]. Furthermore, *A. tumida* can invade other species including stingless bees (Apidae: Meliponini) [24,25], bumblebees (*Bombus* spp.) [26], and solitary bees [27]. *Aethina tumida* can also serve as a vector for the transmission of diseases, including *Paenibacillus larvae* [28,29] and viruses, such as the deformed wing virus and sacbrood virus [30]. *Steinernema carpocapsae* (Weiser, 1955), an entomopathogenic nematode characterized by a broad insecticidal spectrum; facile cultivation; low resistance potential; and safety to humans, livestock, and the environment; has emerged as one of the most promising biological control agents against *A. tumida* [31,32]. When using *S*. *carpocapsae* for control, the best control period is the mature stage when *A. tumida* exists in the soil, and the control effect can reach 100% [9], the mortality rate to the pupa can reach 76–94% [33], and the control effect is the best within the range of 0.90–1.80 m around the beehive [34].

Species distribution models (SDMs) are effective tools for predicting the spatial distribution of suitable habitats. Commonly used SDMs include BIOCLIM, GARP, MaxEnt, CLIMEX, and DOMAIN, which have been extensively applied across diverse research domains [35,36,37]. The MaxEnt model currently ranks among the most widely used species distribution modeling approaches. This model predicts the potential species distribution range based on occurrence records and environmental variables [38,39]. CLIMEX is a semi-mechanistic modeling methodology that emphasizes the ecological and physiological responses of a species. Drawing upon geographical distributions and biological characteristic data, CLIMEX simulates potentially suitable habitat ranges under climatic conditions that optimally correspond to real colonization patterns within documented distribution ranges, thereby predicting potentially suitable habitats within target regions [40]. The use of the MaxEnt and CLIMEX models can provide a more comprehensive niche model analysis, improve the accuracy of predictions, and better meet the challenges of biodiversity conservation and ecological management.

This study used both MaxEnt and CLIMEX models to predict the global potential distribution of the invasive species *A. tumida* and its natural enemy *S. carpocapsae* under current and future climatic conditions. The primary objectives were to (1) identify the key environmental factors influencing the potential geographical distribution of both species; (2) forecast potential distribution patterns and dynamic range shifts under climate change scenarios; and (3) establish a theoretical framework to inform prevention, monitoring, and sustainable management strategies.

## 2. Materials and Methods

### 2.1. Occurrence Data

**Figure 1:**
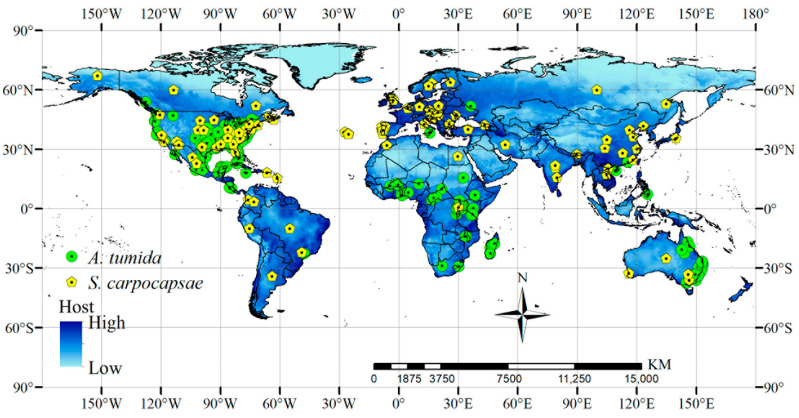
Global geographic distribution records of *A. tumida* (green dots), its natural enemies *S. carpocapsae* (yellow dots) and host suitable habitat from MaxEnt model (blue stripe).

For *A. tumida*, occurrence data were obtained from Global Biodiversity Information Facility (GBIF, available online: https://doi.org/10.15468/dl.fmh8ak, accessed on 16 April 2025) and relevant published literature [12]. For *S. carpocapsae*, GBIF (available online: https://doi.org/10.15468/dl.kcr36u, accessed on 25 November 2025), Centre for Agriculture and Bioscience International (CABI), and previously published literature were used for occurrence data [41,42,43]. Data was preprocessed using the R package dismo (https://github.com/rspatial/dismo, accessed on 25 November 2025) in R 4.5.2. (https://www.r-project.org/, accessed on 25 November 2025) to eliminate duplicate records, geospatially erroneous points, and observations lacking geographic information. To mitigate sampling bias and spatial autocorrelation, the spatial rarefy occurrence tool in the SDM Toolbox 2.5 was employed to thin the dataset by applying a minimum distance threshold of 5 km between retained points [44]. The final modeling dataset comprised 225 occurrence data points for *A. tumida* and 94 for *S. carpocapsae*. In addition, the distribution data of *Apis mellifera* (Linnaeus, 1758), the main host of *A. tumida*, were obtained from GBIF (available online: https://doi.org/10.15468/dl.4hsz5a, accessed on 19 March 2026) to provide critical context for analyzing the relationship between host availability and target species distribution [45] (Figure 1).

### 2.2. Bioclimatic Data

Current bioclimatic variables were obtained from the World Climate Database (http://www.worldclim.org/, accessed on 25 November 2025) at a spatial resolution of 2.5 arc-minutes. This dataset comprised 19 bioclimatic variables (bio1–bio19), representing the minimum, maximum, and mean values of monthly, seasonal, and annual ambient temperatures for the baseline period of 1970–2000 [46] (Appendix A, Table A1). For CLIMEX modeling, climate data were obtained from the CliMond dataset (https://www.climond.org/, accessed on 25 November 2025) with a spatial resolution of 30 arc-minutes based on the Special Report on Emission Scenarios (SRES). This dataset encompasses long-term monthly averages of minimum and maximum temperatures, together with monthly mean relative humidity at 09:00 (RH 0900) and 15:00 (RH 1500), spanning 1961–1990 and centered on 1975 [47].

Future bioclimatic variables were derived from Coupled Model Intercomparison Predicted Phase 6 (CMIP6) models [48]. Three Shared Socioeconomic Pathways (SSPs; SSP126, SSP245, and SSP585) were selected to represent low, medium, and high greenhouse gas emission trajectories, respectively [49], across four future periods: the 2030s (2021–2040), 2050s (2041–2060), 2070s (2061–2080), and 2090s (2081–2100). For the CLIMEX model, the A1B and A2 scenarios were employed for the 2030s, 2050s, 2070s, 2090s, and 2100s, with each time node representing a 20-year average climate [50]. The A1B scenario characterizes rapid global economy integration, diversified and balanced energy development, and the progressive substitution of fossil fuels by non-fossil energy sources as the dominant primary energy component, predicting a global mean surface temperature increase of 1.4–3.8 °C by 2100s (best estimate: 2.4 °C). The A2 scenario depicts a regionally heterogeneous development pattern characterized by sustained global population growth and fragmented, decentralized economic expansion, assuming atmospheric CO_2_ concentrations of 846 ppm and predicting approximately 6 °C warming by the end of this century [51].

**Figure A1:**
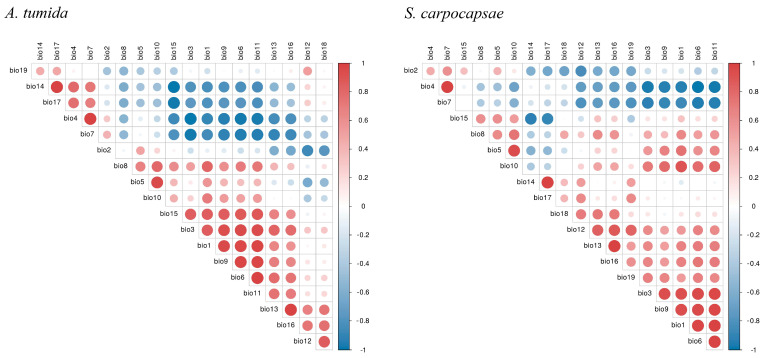
Correlation matrix among environmental variables.

Environmental variables exhibit substantial spatial correlation, which may compromise predictive accuracy and create overfitted models [52]. To address multicollinearity, Pearson’s correlation analysis was performed using IBM SPSS Statistics 27, and variables exhibiting the strongest autocorrelation (|r| > 0.8) were excluded (Appendix B, Figure A1) [53,54]. The final variable set comprised five bioclimatic predictors for *A. tumida* (bio2, bio10, bio11, bio12, and bio18) and five for *S. carpocapsae* (bio2, bio11, bio17, bio18, and bio19).

### 2.3. The MaxEnt Model

The MaxEnt model (3.4.4; https://biodiversityinformatics.amnh.org/open_source/maxent/, accessed on 25 November 2025) uses maximum entropy theory to estimate the potential distributions of species by contrasting occurrence localities against randomly sampled background points. Model parameterization followed established protocols: 75% of occurrence records were selected for model training, with the remaining 25% reserved for independent validation; the regularization multiplier was set to 1; repetitions were fixed at 10 runs; maximum iterations were constrained to 500; and 10,000 random background points were generated. Variable importance was assessed using the jackknife method, and response curves were generated to characterize species–environment relationships. The model output was exported as continuous raster data in a logistic format, with all remaining parameters retained at their default values [55,56]. The results were categorized into four classes using the reclassification tool in ArcGIS 10.8: unsuitable (0–0.2), marginally suitable (0.2–0.4), moderately suitable (0.4–0.6), and highly suitable (0.6–1) [57]. To analyze the spatiotemporal dynamics of species distribution, continuous outputs were converted to binary presence/absence maps using the thresholding tool in the SDM Toolbox, from which range shifts were classified as range expansion, no change, or range contraction [58,59]. The MaxEnt tool in the “SDM Tools” module was used to calculate the predicted expansion and contraction area for the future.

Model performance was evaluated using the area under the receiver operating characteristic curve (AUC) and true skill statistics (TSSs) [55]. The AUC ranged from 0 to 1 and integrates sensitivity and specificity. Values of >0.9 indicate excellent, 0.8–0.9 good, and <0.7 poor performance [60,61]. TSSs were calculated using the validation dataset and were not affected by the size of the validation dataset, ranging from −1 to 1, with values approaching 1 indicating optimal agreement between predictions and observations. Values of >0.75 denote excellent model accuracy [62].

**Figure A2:**
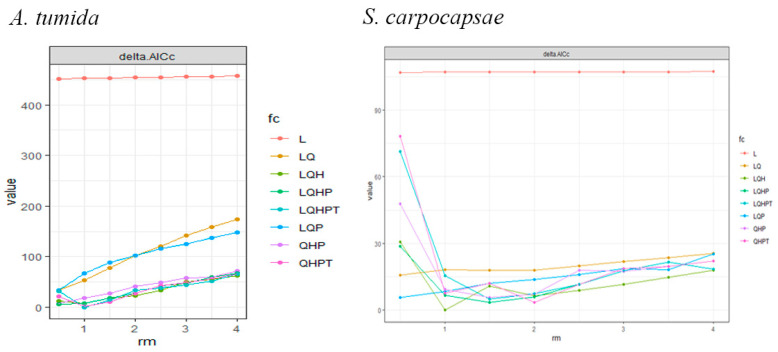
The AICc value of the parameter combination calculated using ENMeval.

The predictive accuracy of MaxEnt is primarily governed by the regularization multiplier (RM) and feature combination (FC). The R package ENMeval 2.0.5.2 (https://jamiemkass.github.io/ENMeval/articles/ENMeval-2.0-vignette.html, accessed on 25 November 2025) was used to optimize these parameters and mitigate overfitting [63]. The evaluated feature classes were linear (L), product (P), quadratic (Q), threshold (T), and hinge (H) functions [64]. Eight FC combinations were assessed as candidate models: L, LQ, LQH, LQHP, LQHPT, LQP, QHP, and QHPT. The RM varied from 0.5 to 4.0 in incremental 0.5-unit steps [65]. Model selection was based on the Akaike information criterion corrected for small sample sizes (AICc), with the optimal model exhibiting the lowest delta AICc (ΔAICc = 0) [66,67]. Following optimization, the selected parameters were FC = LQHPT and RM = 1 for *A. tumida* and FC = LQH and RM = 1 for *S. carpocapsae*, both with ΔAICc = 0 (Appendix B, Figure A2).

### 2.4. The CLIMEX Model

CLIMEX v.4.0 (Hearne Scientific Software, Australia) was implemented using the “compare locations” function to construct simulation models [68,69]. Key biological parameters incorporated included: developmental temperature thresholds (DV0, DV1, DV2, and DV3), soil moisture thresholds (SM0, SM1, SM2, and SM3), degree-days per generation (PDD), cold stress parameters (threshold temperature, TTCS; accumulation rate, THCS), heat stress parameters (threshold temperature, TTHS; accumulation rate, THCS), dry stress parameters (threshold temperature, SMDS; accumulation rate, HDS), and wet stress parameters (threshold soil moisture, SMWS; accumulation rate, HWS) [70]. These parameter settings were based on relevant data from the literature [21,71,72,73] and templates provided by CLIMEX software. The parameter values were continuously adjusted until they matched the actual situation, and the final parameter values were determined (Appendix A, Table A2). A suitable visualization range was drawn using the ArcGIS software [74]. Model outputs were expressed as ecoclimatic index (EI) values, ranging from 0 to 100, where EI = 0 indicates that the region is unsuitable for species survival and an EI approaching 100 indicates that the climate and environment in the region are more suitable [75,76]. The EI value was determined by the stress index (SI), annual growth index (GI), and stress interaction index (SX). This led to a comprehensive EI formula: EI = SI × GI × SX [77]. Values were classified as unsuitable (EI = 0), marginally suitable (0 < EI < 10), moderately suitable (10 < EI < 20), and highly suitable (EI > 20) [78].

### 2.5. Ensemble Map

In the current climate prediction map, the probability output of MaxEnt and EI of CLIMEX were converted into binary layers in the presence and absence of geographic space. The probability output of MaxEnt and the EI of CLIMEX were binarized: MaxEnt used a threshold of 10 percentile presence in the training data, while CLIMEX used a threshold of EI > 0 to generate binary distribution layers. The consistency between the models in geographical space was evaluated through the spatial overlay analysis of two sets of binary map layers [79]. The performance of the CLIMEX model was evaluated by comparing the potential distribution areas predicted by the model with the actual observation records of the species. The EI values of the model were compared with the actual observed ecological data to confirm whether the model could correctly simulate the ecological conditions of the species. In species distribution modeling, coverage is typically calculated by dividing the predicted suitable area by the total area of the study area (usually expressed as a percentage).

## 3. Results

### 3.1. Model Accuracy Evaluation and Host Availability

**Figure A3:**
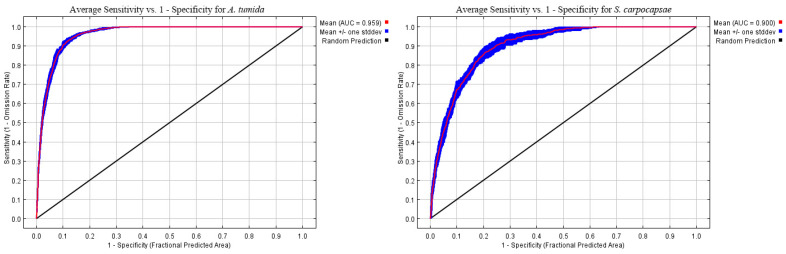
The AUC curves in the distribution model.

Following ten replicate runs, the MaxEnt models demonstrated robust predictive performance, with *A. tumida* achieving AUC = 0.959 and TSS = 0.815 and *S. carpocapsae* AUC = 0.900 and TSS = 0.842. These results indicated excellent model accuracy and high reliability for predicting suitable habitats (Appendix B, Figure A3).

**Figure A4:**
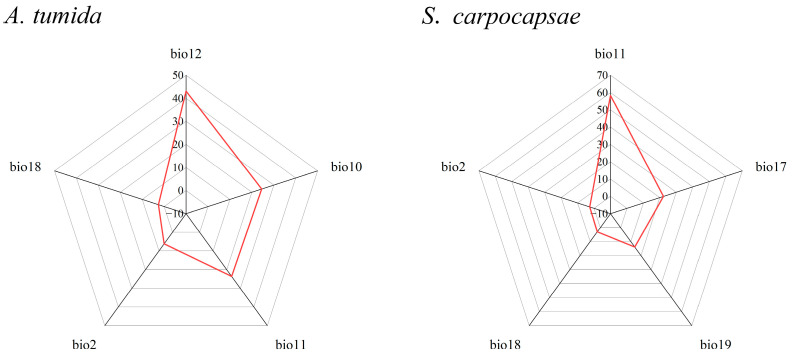
Relative contribution of each environmental variable to the potential distribution. The red line represents the percentage value of each environmental variable.

**Figure A5:**
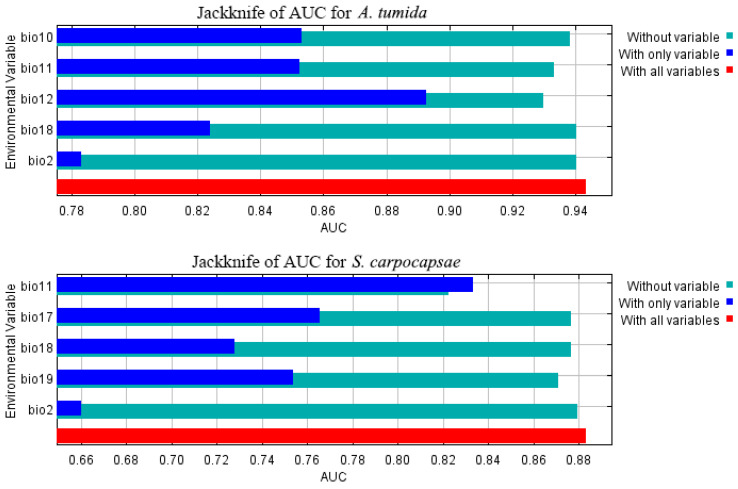
The relative predictive power of different environmental variables based on the jackknife of regularized training gain in MaxEnt models.

**Figure A6:**
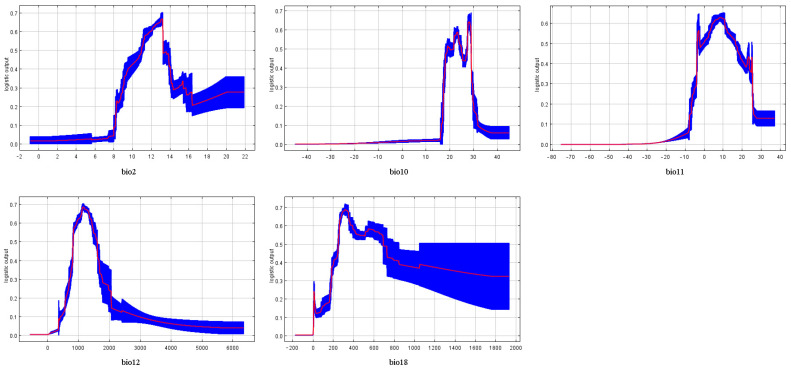
Response curve of the environmental variables of *A. tumida*.

**Figure A7:**
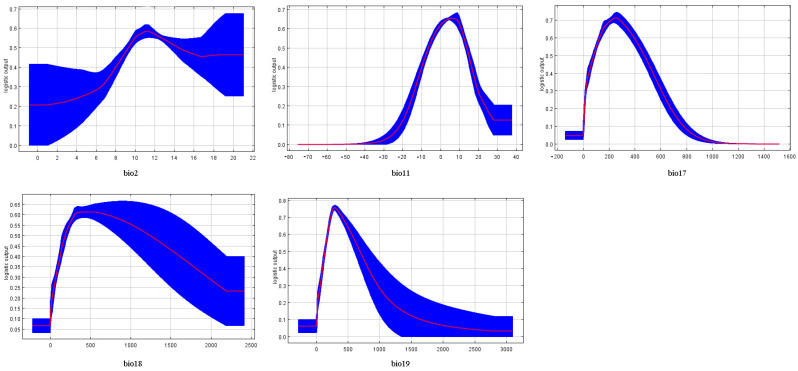
Response curve of the environmental variables of *S. carpocapsae*.

The percentage contribution revealed that annual precipitation (bio12, 43.1%), mean temperature of the warmest quarter (bio10, 24.5%), and mean temperature of the coldest quarter (bio11, 23.6%) were the predominant determinants of *A. tumida* distribution, which collectively accounted for 91.2% of the explained variance. For *S. carpocapsae*, the most important environmental factors were the mean temperature of the coldest quarter (bio11, 58.3%), precipitation of the driest quarter (bio17, 22.2%), and precipitation of the coldest quarter (bio19, 13.9%), contributing 94.4% cumulatively (Appendix B, Figure A4). Jackknife regularization tests corroborated these findings, identifying bio12 and bio11 as the variables with the highest predictive power for *A. tumida* and *S. carpocapsae*, respectively, both exhibiting training gains exceeding 0.82 (Appendix B, Figure A5). The response curves illustrated species–environmental variables with high clarity: optimal conditions for *A. tumida* occurred at bio2 = 8–15 °C, bio10 = 18–30 °C, bio11 = −10–25 °C, bio12 peaking at 1010 mm (suitability = 0.7), and bio18 = 0–800 mm (Appendix B, Figure A6). For *S. carpocapsae*, suitable ranges occurred at bio2 = 8–16 °C, bio11 = −10–20 °C, bio17 = 0–1000 mm, bio18 = 0–800 mm, and bio19 = 0–600 mm (Appendix B, Figure A7).

### 3.2. Potential Distribution of A. tumida and Host Availability

It is worth noting that the potential distribution of the host mostly covers the distribution range of *A. tumida*. The MaxEnt results indicate that the host is widely distributed across all continents, particularly over Europe and North America (Figure 1).

### 3.3. Potential Distributions Under Climate Conditions Using MaxEnt

**Figure 2:**
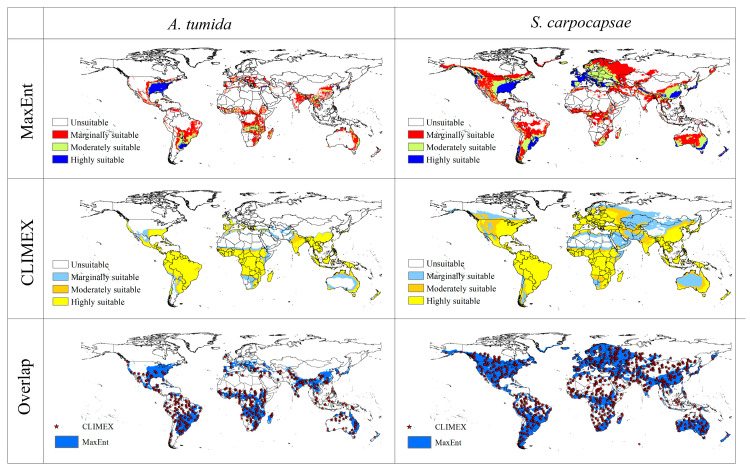
The potential global distribution habitat of *A. tumida* and *S. carpocapsae* under current climate scenarios using MaxEnt and CLIMEX models. Combined prediction maps intersected with the predicted suitable habitat from the MaxEnt and CLIMEX models under current climate conditions.

**Figure 3:**
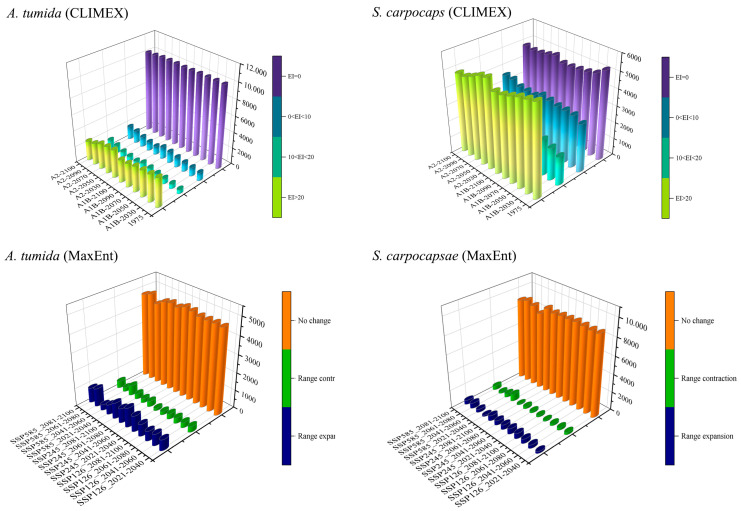
Changes in the potential distribution habitat for *A. tumida* and *S. carpocapsae* under future climatic scenarios (10^4^ km^2^).

**Figure 4:**
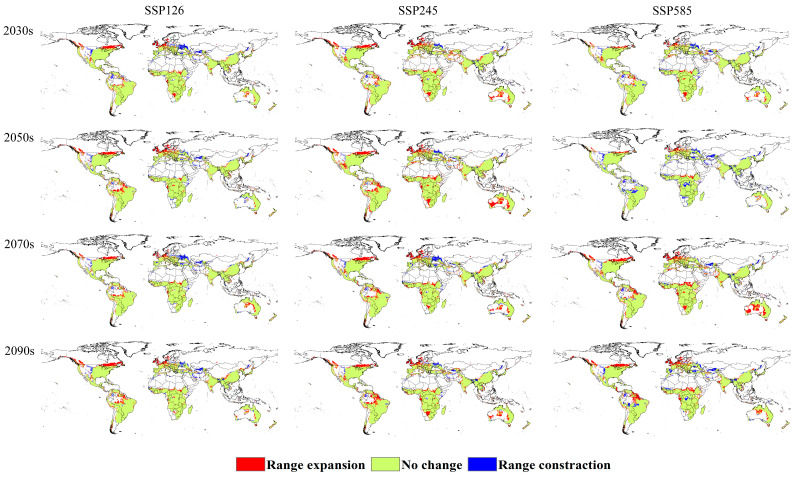
Changes in habitat of the potential distribution of *A. tumida* under future climate scenarios, using MaxEnt model.

**Figure 5:**
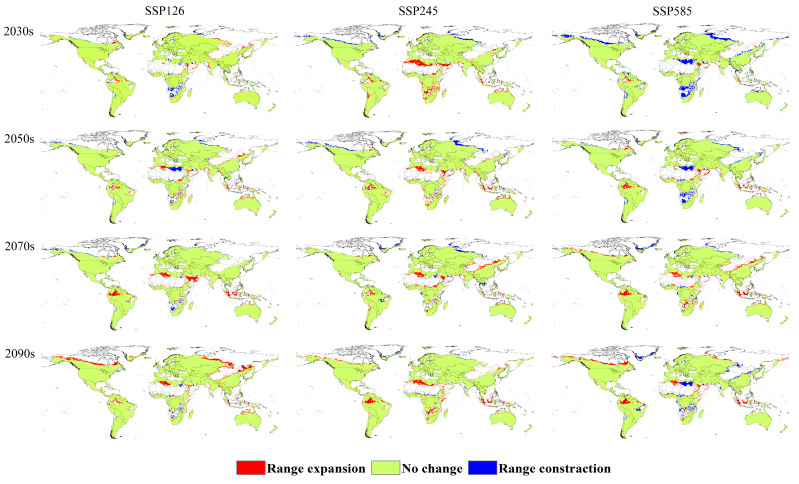
Changes in habitat of the potential distribution of *S. carpocapsae* under future climate scenarios, using MaxEnt model.

The MaxEnt model was used to predict potential shifts in the global geographic distributions of *A. tumida* and *S. carpocapsae* under current and future SSP scenarios (SSP126, SSP245, and SSP585) across four different periods (2030s, 2050s, 2070s, and 2090s) (Figure 2, Figure 3, Figure 4 and Figure 5).

Under current climatic conditions, highly suitable habitats for *A. tumida* occupied 3.75 million km^2^ (13.03% of the total suitable area), concentrated predominantly over the United States of America. Moderately suitable habitats encompassed 8.39 million km^2^ (29.15%), distributed across eastern Argentina, southern Brazil, Angola, Zambia, and other parts of southern Africa. Marginally suitable habitats comprised 16.64 million km^2^ (57.82%), distributed across the central regions of South America, southern Africa, and southern Asia (Figure 2 and Figure 3). Under future climatic scenarios, divergent distributional trajectories emerge: SSP245 (2050s) and SSP585 (2070s) witness a marked expansion across North America, Europe, and central Australia, whereas SSP585 (2050s) showed pronounced contractions across Asia, Africa, and South America (Figure 4).

For *S. carpocapsae*, highly suitable habitats totaled 10.09 million km^2^ (14.96% of the total suitable area), with distributions over southern China, the United States and parts of Europe. Moderately suitable habitats covered 21.92 million km^2^ (32.49%), concentrated over the United States, portions of South America, Europe, and Asia, whereas marginally suitable habitats extended across 35.46 million km^2^ (52.56%), encompassing all continents (Figure 2 and Figure 3). Projections into the future revealed complex spatiotemporal dynamics: substantial range expansion into Asia, North America, and Africa is predicted under SSP126 (2090s), SSP245 (2030s), and SSP585 (2070s), yet substantial contractions are predicted under SSP585 by the 2030s (Figure 5).

### 3.4. Potential Distributions Under Climate Conditions Using CLIMEX

**Figure 6:**
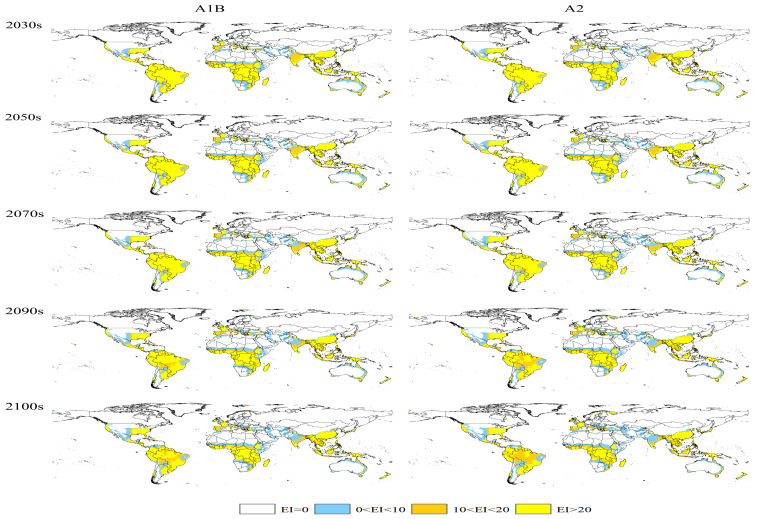
Changes in habitat of the potential distribution of *A. tumida* under future climate scenarios, using CLIMEX model.

**Figure 7:**
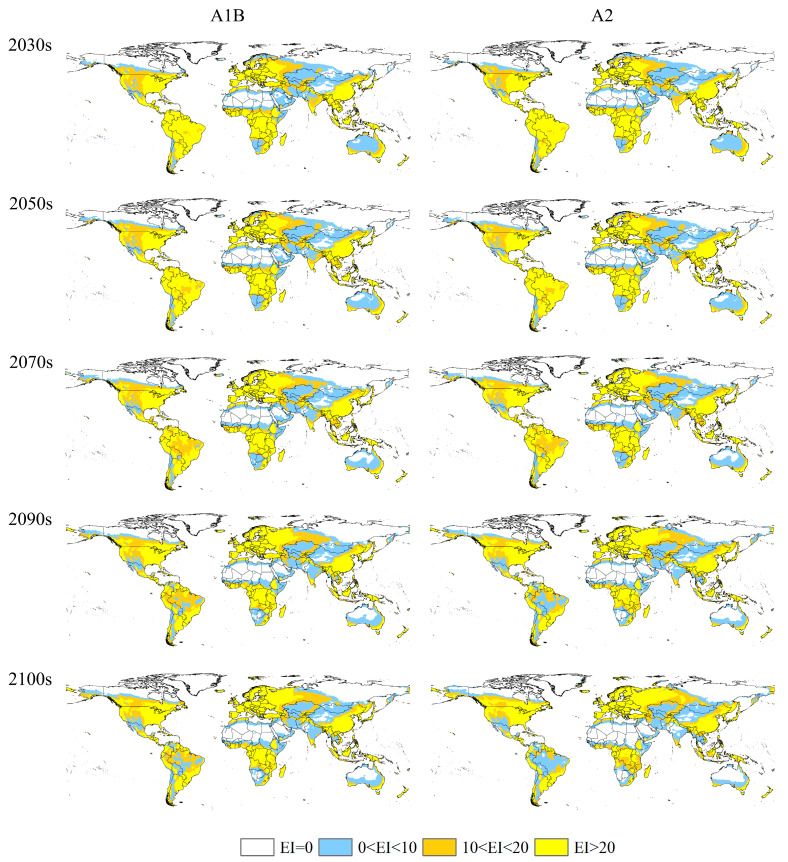
Changes in habitat of the potential distribution of *S. carpocapsae* under future climate scenarios, using CLIMEX model.

CLIMEX was used to predict the potential global distribution shifts for *A. tumida* and *S. carpocapsae* under the current climate and two climate scenarios (A1B, A2) across five temporal horizons (2030s, 2050s, 2070s, 2090s, and 2100s) (Figure 2, Figure 3, Figure 6 and Figure 7).

Under the current climatic conditions, potentially suitable habitats of *A. tumida* encompass South America, southern Asia, and the majority of Africa. Under scenarios A1B and A2, the total suitable habitat exhibits incremental expansions of 0.94% and 2.38%, respectively, by the 2100s. However, this aggregate stability masked substantial internal reconfiguration: highly suitable habitats contracted markedly by 22.73% (A1B) and 33.08% (A2), whereas moderately suitable habitats expanded by 78.65% (A1B) and 131.96% (A2) in the 2100s. Similarly, marginally suitable habitats were projected to increase by 45.78% (A1B) and 62.76% (A2) by the 2100s (Figure 2, Figure 3 and Figure 6).

Under current climatic conditions, suitable habitats for *S. carpocapsae* are distributed across all continents. Climate change predictions indicate a substantial restructuring of this distribution pattern. Under both the A1B and A2 climate scenarios, highly suitable habitats are predicted to contract substantially, whereas moderately and marginally suitable habitats are expected to expand. Specifically, by the 2100s, highly suitable habitats are expected to decrease by 23.42% (A1B) and 22.54% (A2). By contrast, moderately suitable habitats exhibited a pronounced expansion of 91.74% under A1B (2100s) and 86.03% under A2 (2090s). Similarly, marginally suitable habitats are projected to increase by 10.29% (A1B) and 21.27% (A2) by the 2100s (Figure 2, Figure 3 and Figure 7).

### 3.5. Combined Prediction Maps of A. tumida and S. carpocapsae Under Current Climate Conditions

To enhance predictive robustness, the outputs from MaxEnt and CLIMEX were integrated under current climatic conditions, with spatial overlap between model predictions interpreted as high-confidence suitable habitats. Both models predicted pantropical and temperate distribution potential across all continents. Notably, the CLIMEX predictions encompassed the entire spatial domain predicted by MaxEnt (Figure 2).

## 4. Discussion

This study used MaxEnt and CLIMEX models to predict potentially suitable habitats for *A. tumida* and *S. carpocapsae* under current and future climate change scenarios. Following parameter optimization (FC = LQHPT, RM = 1 for *A. tumida*; FC = LQH, RM = 1 for *S. carpocapsae*), both models achieved AUC and TSS values greater than 0.8, indicating good predictive accuracy and high reliability [80,81]. The relative importance of bioclimatic variables differed between the species. The distribution of *A. tumida* was predominantly constrained by annual precipitation (bio12), the mean temperature of the warmest quarter (bio10) and the mean temperature of the coldest quarter (bio11), corroborating previous findings [12]. Conversely, *S. carpocapsae*’s suitable habitats were principally governed by the mean temperature of the coldest quarter (bio11) and the precipitation of the driest quarter (bio17). Temperature and precipitation, thus, emerged as limiting climatic factors for the species, supporting the established ecological theory [82].

Temperature exerts direct physiological control over *A. tumida*’s developmental rate and survival across its life stages. The lower thermal thresholds for egg, larval, and pupal development range from 10.0 to 13.5 °C [71], with temperatures exceeding 35 °C severely compromising egg viability. Under favorable thermal regimes, *A. tumida* can complete up to six generations per year. Pupal survival and developmental duration are temperature-dependent, with optimal conditions prolonging development and enhancing survival [72]. Notably, *A. tumida* has a remarkable cold tolerance and is capable of overwintering within hives at −40 °C, though it cannot persist outside hive structures in the cold, and its reproduction occurs in the northern summer [71,83,84]. Humidity further modulates population dynamics: a relative humidity below 50% facilitates egg hatching, and larval-to-pupal development typically requires 10–14 days, with a pupation rate of 92–98% recorded in moist soil [21]. As a thermophilic and hygrophilic pest, *A. tumida* is poised to expand its distributional range under global warming scenarios, posing a persistent threat to apicultural systems worldwide [85]. Meanwhile, *S. carpocapsae* is most effective in the temperature range of 22–28 °C (72–82 °F) [73].

Under the current climatic conditions, both models indicate that suitable habitats for *A. tumida* are concentrated in South America, southern Africa, and southern Asia, whereas *S. carpocapsae* exhibited a broader, near-cosmopolitan potential distribution. A comparative analysis revealed that CLIMEX predictions encompassed more extensive suitable ranges than MaxEnt for both species, with CLIMEX outputs effectively subsuming the MaxEnt predictions.

Both the CLIMEX and MaxEnt models revealed an overall trend toward high-latitude migration when predicting the future distribution of the species, but they showed considerable differences in the range of potentially suitable areas. This divergence is mainly attributed to the inherent differences in the algorithmic mechanisms. As a correlation model, MaxEnt relies on the relationship between species occurrence records and environmental variables, which can include factors such as soil and altitude. The predicted results are often limited by the environmental characteristics of the current distribution point and, thus, exhibit strong conservatism. By contrast, as a semi-mechanistic model, CLIMEX explicitly simulates the tolerance limits of species to climate by integrating distribution data and ecological physiological parameters, such as cold, hot, dry, and wet stress indices. This semi-mechanistic-based prediction enabled CLIMEX to predict a wider range, whereas MaxEnt limited its prediction to areas with similar known occurrences. Integrating these two models can effectively reduce the uncertainty caused by a single algorithm while simultaneously improving the confidence of the predictions [75].

*Aethina tumida*, a nest pest of the western honey bee *Apis mellifera*, is indigenous to Europe, Africa, and the Middle East but has achieved cosmopolitan distribution through anthropogenic dispersal [83]. Intensified international trade and bee stock exchanges have facilitated the range expansion of *A. tumida*, causing severe damage to apicultural operations [14]. Beyond structural hive degradation, infestations precipitate colony depopulation and cause substantial economic losses. *Steinernema carpocapsae*, a natural enemy of *A. tumida*, demonstrates considerable potential for biological control applications by effectively suppressing pest populations. Their use can provide critical technical support for sustainable and environmentally benign management strategies [34].

This study incorporated 19 bioclimatic variables to predict potentially suitable habitats for *A. tumida* and its natural enemy, *S. carpocapsae*. In spatial analysis, we observed that the sampling work was unbalanced, with areas with high recording density concentrated in certain regions (such as agricultural monitoring points or easily accessible areas), which may lead to spatial autocorrelation and overfitting in specific regions. In addition, edaphic factors such as soil temperature and moisture may significantly influence species distribution [84]. The models did not account for critical biotic and abiotic factors, such as interspecific competition, anthropogenic disturbance, and dispersal barriers, which constrained their ability to explain the actual invasion dynamics. Beehive microclimates and other environmental features from macroclimatic models reduce predictive precision. The predictive performance of the CLIMEX model mainly depends on biological parameters and climate data. Biological parameters are usually collected from the literature and adjusted through calibration. Although the calibrated model can reproduce the historical distribution of species well, there are still uncertain factors, such as stress accumulation rate, which often have subjective judgments. The data provided by different literature or experiments vary greatly, which can easily lead to significant deviations in the final results [59]. Therefore, the conclusion of this study focuses on presenting the potential range of predictions rather than absolute precision values.

Future research should integrate species-specific biological characteristics, beehive microclimates, and wild habitat characteristics, incorporating environmental covariates such as soil physicochemical properties and topographic factors, while supplementing biotic and abiotic factors to include interspecific competition and human activities. Such comprehensive model optimization would provide a robust theoretical foundation for biosecurity management.

By integrating current and future distribution dynamics, focusing on the ecological interactions between pests and nematodes informs evidence-based management strategies. Given the versatile nature of nematodes, their range depends not only on the presence of pests but also on the availability of alternative hosts and specific abiotic constraints, particularly soil moisture and temperature conditions required for their free life stage. Therefore, although the distribution of pests may define potential interactions, the wider environmental tolerance and polyphagous behavior of nematodes may enable them to live in a broader basic ecological niche.

Global agricultural systems exhibit substantial dependence on bee-mediated pollination services, particularly for oilseed crops, forage plants, and fruit and vegetable production, thereby conferring significant economic and ecological value on bees. Invasive pests are disseminated across international borders via trade and transportation networks and pose a severe threat to apicultural industries and ecological security. Port quarantine protocols should prioritize high-risk vectors, including bee products, wooden packaging materials, and soil-adhered horticultural commodities, with concomitant strengthening of access management and early warning systems. For regions where establishment has already occurred, adaptive zonal establishment should be implemented, including enhanced monitoring and emergency response capacity in highly suitable habitats, establishing buffer zones in peripheral habitats, and developing transregional joint prevention and control mechanisms.

## 5. Conclusions

The potentially suitable distributions of *A. tumida* are predominantly concentrated in South America, Africa, and Asia. Under scenarios of continued global environmental change and intensified anthropogenic activities, this species exhibits a pronounced range-expansion trajectory. Given its substantial invasive potential and the widespread distribution of hosts, prevention and control measures must be strengthened to reduce threats to ecosystems and agricultural production systems. Simultaneously, using *S. carpocapsae* as a biological control of *A. tumida* is promising, as it is distributed worldwide owing to its broad environmental tolerance and effective dispersal mechanisms, which underscores the need for sustained monitoring and targeted management strategies for this biological control agent.

## Data Availability

All relevant data are within the paper.

## Abbreviations

The following abbreviations are used in this manuscript:

SDMs	Species distribution models
CMIP6	Coupled Model Intercomparison Predict Phase 6
SSP	Shared Socioeconomic Pathway
AUC	Area under the ROC curve
TSS	True skill statistic

## Appendix A

**Table A1:** Environmental variables used in this study.

Variable	Description	
Bio1	Annual Mean Temperature	°C
Bio2	Mean Diurnal Range	°C
Bio3	Isothermality	°C
Bio4	Temperature Seasonality	°C
Bio5	Max. Temperature of Warmest Month	°C
Bio6	Min. Temperature of Coldest Month	°C
Bio7	Temperature Annual Range	°C
Bio8	Mean Temperature of Wettest Quarter	°C
Bio9	Mean Temperature of Driest Quarter	°C
Bio10	Mean Temperature of Warmest Quarter	°C
Bio11	Mean Temperature of Coldest Quarter	°C
Bio12	Annual Precipitation	mm
Bio13	Precipitation of Wettest Month	mm
Bio14	Precipitation of Driest Month	mm
Bio15	Precipitation Seasonality	%
Bio16	Precipitation of Wettest Quarter	mm
Bio17	Precipitation of Driest Quarter	mm
Bio18	Precipitation of Warmest Quarter	mm
Bio19	Precipitation of Coldest Quarter	mm

**Table A2:** CLIMEX modeling parameters.

Parameter	Meaning of Parameter	*A. tumida*	*S. carpocapsae*
DV0	Limiting low temperature	8	−10
DV1	Lower optimum temperature	10	20
DV2	Upper optimum temperature	32	30
DV3	Limiting high temperature	45	42
SM0	Limiting low soil moisture	0.1	0
SM1	Lower optimal soil moisture	1	0.5
SM2	Upper optimal soil moisture	1.5	1.5
SM3	Limiting high soil moisture	2.5	2
TTCS	Cold stress temperature threshold	0	−10
THCS	Cold stress temperature rate	−0.001	−0.0001
TTHS	Heat stress temperature threshold	50	42
THHS	Heat stress temperature rate	0.01	0.0005
SMDS	Dry stress temperature threshold	0.2	0.1
HDS	Dry stress accumulated rate	−0.001	−0.0001
SMWS	Wet stress temperature threshold	10	10
HWS	Wet stress accumulated rate	0.001	0.05
PDD	Degree-days per generation	400	510
